# Body Mass Index and Body Fat Percentage Are Associated with Clinical Outcomes in Patients Receiving PD-1/PD-L1 Inhibitors: A Prospective Exploratory Study

**DOI:** 10.3390/jpm16090457

**Published:** 2026-08-30

**Authors:** Ángela Morell, Alberto Morell, Ainhoa Aranguren, Jacobo Rogado, Miguel Sampedro, Rebeca Mondéjar, Ramón Colomer, M. Paz Lorenzo, Esther Ramírez

**Affiliations:** 1 Faculty of Medicine, Universidad San Pablo-CEU, CEU Universities, Urbanización Montepríncipe, Boadilla del Monte, 28668 Madrid, Spain; 2 Hospital Pharmacy Service, Hospital Universitario de la Princesa, 28006 Madrid, Spain; albertomorell04@gmail.com (A.M.); ainhoa.aranguren@salud.madrid.org (A.A.); 3 Instituto de Investigación Sanitaria del Hospital Universitario de la Princesa, 28006 Madrid, Spain; rebeca.mondejar@salud.madrid.org (R.M.); rcolomer@seom.org (R.C.); 4 Medical Oncology Department, Hospital Universitario de la Princesa, 28006 Madrid, Spain; jacobo.rogado@gmail.com; 5 Department of Endocrinology and Nutrition, Hospital Universitario de la Princesa, 28006 Madrid, Spain; miguelantonio.sampedro@salud.madrid.org; 6 Cátedra de Medicina Personalizada de Precisión, Universidad Autónoma de Madrid, 28006 Madrid, Spain; 7 Centro de Metabolómica y Bioanálisis (CEMBIO), Facultad de Farmacia, Universidad San Pablo-CEU, CEU Universities, Urbanización Montepríncipe, Boadilla del Monte, 28668 Madrid, Spain

**Keywords:** immune checkpoint inhibitors, body composition, bioelectrical impedance analysis

## Abstract

**Background/Objectives**: Immunotherapy using immune checkpoint inhibitors has transformed the treatment of multiple solid tumours. However, the clinical response to programmed death receptor (PD-1) and its ligand (PD-L1) inhibitors remains heterogeneous. We have previously shown that host-related factors such as body mass index (BMI) may modulate the efficacy of immunotherapy. The aim of this study was to determine whether body composition, measured with bioelectrical impedance analysis (BIA) before treatment initiation, is a prognostic factor for survival outcomes in patients treated with PD-1/PD-L1 inhibitors, independently of tumour type and stage. **Methods**: In this prospective, single-centre observational study, patients with solid tumours underwent baseline body composition assessment using multifrequency bioelectrical impedance analysis (BIA) before initiation of PD-1/PD-L1 inhibitor therapy. Overall survival (OS), progression-free survival (PFS), and treatment-related adverse events were evaluated using Kaplan–Meier analyses and multivariable Cox regression models adjusted for tumour stage and metastatic status. **Results**: A total of 80 patients with solid tumours were included in the study. The median age was 67 years, and 48 (60.0%) were male. Higher BMI (≥25 kg/m^2^) and elevated body fat percentage (%BF) were associated with more favourable survival outcomes. After adjustment for tumour stage and metastatic status, BMI ≥ 25 kg/m^2^ was associated with a lower risk of death (HR 0.286, 95% CI 0.087–0.941; *p* = 0.039). Similar results were observed for elevated %BF (HR 0.289, 95% CI 0.087–0.963; *p* = 0.036). Neither BMI nor %BF was significantly associated with the occurrence of confirmed immune-related adverse events. **Conclusions**: Baseline BMI and body fat percentage were associated with survival outcomes in this exploratory cohort of patients receiving PD-1/PD-L1 inhibitors. These findings should be considered exploratory and hypothesis-generating and require confirmation in larger, tumour-specific prospective studies.

## 1. Introduction

Immune checkpoint inhibitors (ICIs) targeting programmed cell death protein-1 (PD-1) and its ligand (PD-L1) have revolutionised the treatment of several advanced solid tumours, providing durable responses and prolonged survival in a subset of patients. However, substantial interindividual variability remains in both treatment efficacy and toxicity, highlighting the need to identify patient-related factors that may predict treatment response and the risk of developing immune-related adverse events [1,2,3].

Current predictive biomarkers, such as PD-L1 expression and tumour mutational burden, only partially explain the heterogeneity observed in clinical responses to immunotherapy [1]. Increasing evidence suggests that patient-related characteristics, including nutritional status, body composition, systemic inflammation, and metabolic profile, may also influence immune function and treatment outcomes. Consequently, these factors have attracted growing interest as potential tools for improving patient stratification and advancing a more personalised approach to cancer care [4,5].

Among these factors, obesity has emerged as an area of particular interest. Although traditionally associated with chronic inflammation and adverse health outcomes, several observational studies have reported improved survival among overweight and obese patients receiving ICIs compared with those with normal or low body mass index (BMI), a phenomenon commonly referred to as the obesity paradox. Nevertheless, the biological mechanisms underlying this association remain poorly understood, and it is still uncertain whether BMI adequately reflects the body composition characteristics that may influence the response to immunotherapy [6,7,8,9].

However, evidence regarding the obesity paradox remains inconsistent [10]. An additional challenge in interpreting the association between BMI and outcomes is the potential influence of reverse causality. Patients with lower BMI may represent a subgroup with cancer cachexia, poorer nutritional status, reduced physiological reserve, or more advanced systemic disease [10,11]. Therefore, observations linking higher BMI to improved outcomes should not be interpreted as evidence of a direct beneficial effect of obesity.

BMI is a simple and widely used anthropometric measure; however, it cannot differentiate adipose tissue from lean mass and may not accurately reflect body composition in patients with cancer [12]. Consequently, patients with similar BMI values may present markedly different body composition profiles.

Body fat percentage (%BF) is considered a key complementary indicator because it provides a more direct estimate of adiposity and allows differentiation between fat mass and lean tissue, thereby overcoming some of the limitations of BMI [12].

In this context, bioelectrical impedance analysis (BIA) has emerged as a rapid, non-invasive, and easily applicable technique for body composition assessment, providing additional information on fat mass, skeletal muscle mass, and body water distribution. Compared with BMI, BIA allows a more comprehensive characterisation of the nutritional and metabolic status of patients who are candidates for immunotherapy [12,13,14].

Although several studies have demonstrated that body composition may influence clinical outcomes in patients receiving immunotherapy, the available evidence remains heterogeneous and, in some cases, contradictory. Furthermore, most previous studies have relied on conventional anthropometric measures or imaging techniques, whereas evidence from prospective studies using BIA remains limited [13,14]. Moreover, the impact of adiposity on immunotherapy outcomes may vary according to tumour type, treatment regimen, and patient-related characteristics, highlighting the need for further investigation in prospective real-world cohorts.

Given the limited and sometimes contradictory evidence available, prospective studies evaluating body composition in real-world populations remain necessary to clarify the potential prognostic value of adiposity during treatment with immune checkpoint inhibitors [10,14].

The aim of this prospective observational study was to evaluate whether BMI and body fat percentage determined by BIA, assessed at baseline before initiation of PD-1/PD-L1 inhibitor therapy, constitute a prognostic factor for 1-year clinical outcomes, including overall survival and progression-free survival, independently of tumour type and stage, in patients with solid tumours treated with PD-1/PD-L1 inhibitors, as well as their association with treatment-related toxicity. We hypothesised that body composition assessment could provide additional prognostic information beyond conventional anthropometric measures and contribute to a more personalised evaluation of patients eligible for immunotherapy.

## 2. Materials and Methods

### 2.1. Study Design and Participants

A prospective, longitudinal, single-centre observational study was conducted between March 2022 and August 2024 at Hospital Universitario de La Princesa (Madrid, Spain). The aim of the study was to evaluate whether baseline body composition is a prognostic factor for survival outcomes in patients with solid tumours treated with PD-1/PD-L1 inhibitors, independently of tumour type and stage.

Adult patients (≥18 years) with solid tumours who were scheduled to initiate treatment with PD-1/PD-L1 inhibitors, either as monotherapy or in combination with other antineoplastic therapies, were eligible for inclusion. All participants provided written informed consent before enrolment. Patients who were unable to maintain an upright standing position or who carried implantable electronic devices that could interfere with BIA, such as pacemakers or implantable cardioverter-defibrillators, were excluded. This study was approved by the Research Ethics Committee for Medicines of Hospital Universitario de La Princesa (approval code: 4652) and was conducted in accordance with the principles of the Declaration of Helsinki and current data protection regulations.

### 2.2. Data Collection

Demographic and clinical variables were collected for each patient, including age, sex, weight, BMI, tumour stage, comorbidities and previous treatments, as well as data related to immunotherapy (type of drug, dose and duration). Adverse events were retrieved from the electronic medical records and classified according to version 5.0 of the Common Terminology Criteria for Adverse Events (CTCAE), which categorises events into five grades of severity (grades 1–5).

All PD-1/PD-L1 inhibitors were administered according to approved indications and standard dosing schedules. Treatment line, PD-L1 expression, ECOG performance status and disease burden were not systematically available for all patients and therefore could not be incorporated into the multivariable analyses.

### 2.3. Body Composition Assessment

Body composition was assessed using a segmental multifrequency bioelectrical impedance analyser (InBody 770; InBody Co., Ltd., Seoul, Republic of Korea). This device uses direct segmental multifrequency bioelectrical impedance technology and an eight-electrode system to independently assess the composition of the trunk and limbs. Multiple electrical frequencies are applied to estimate tissue resistance and reactance and to differentiate intracellular and extracellular water compartments, improving the accuracy of fat mass and lean body mass measurements. The device also provides reference values adjusted for age, sex, weight, and height.

The parameters recorded included body weight, body mass index (BMI), body fat percentage (%BF), total fat mass, visceral fat, skeletal muscle mass, fat-free mass, total body water, intracellular water, extracellular water, and phase angle. BIA was selected because it provides a rapid, non-invasive, standardised, and clinically accessible assessment of body composition beyond conventional BMI measurements.

Measurements were performed by the investigator during routine oncology outpatient visits, following the manufacturer’s standardised protocol, with patients in a fasting state, without prior strenuous physical activity, wearing light clothing and without metallic objects, in a standing position. Body composition assessment was performed prior to initiation of PD-1/PD-L1 inhibitor therapy.

Body mass index (BMI) was calculated using the standard formula (weight/height^2^), categorised according to the World Health Organization classification. Body fat percentage (%BF) categories were established according to the age- and sex-specific cut-off values proposed by Gallagher et al. [15].

### 2.4. Study Outcomes

The primary outcomes were overall survival (OS) and progression-free survival (PFS). OS was defined as the time from treatment initiation to death from any cause. PFS was defined as the time from treatment initiation to documented clinical or radiological disease progression or death from any cause, whichever occurred first. Survival outcomes were evaluated at 12 months from the baseline body composition assessment.

Given the limited number of progression events observed during follow-up, an exploratory analysis based on 1-year progression-free status was additionally performed.

### 2.5. Statistical Analysis

Statistical analyses were performed using IBM SPSS Statistics version 25.0 (IBM Corp., Armonk, NY, USA). Continuous variables are presented as means and standard deviations or medians and interquartile ranges, according to their distribution, whereas categorical variables are expressed as frequencies and percentages. Comparisons between categorical variables were performed using the chi-square test or Fisher’s exact test, as appropriate.

Survival analyses were performed using the Kaplan–Meier method, and differences between groups were assessed using the log-rank test. Cox proportional hazards regression models were constructed to evaluate the association between body composition parameters and survival outcomes. Multivariable models were adjusted for tumour stage and metastatic status, which were selected a priori because of their established clinical relevance, their availability across the entire cohort, and because the inclusion of a larger number of covariates in a relatively small sample could increase the risk of model overfitting.

The correlation between BMI and %BF was assessed using Pearson’s and Spearman’s correlation coefficients, both for the continuous variables and for their respective dichotomised categories, to evaluate the degree of overlap between the two measures of adiposity. To further evaluate whether %BF provided prognostic information beyond BMI, an additional exploratory multivariable Cox regression model including both BMI category and %BF category simultaneously, adjusted for tumour stage and metastatic status, was performed for overall survival and progression-free survival.

Associations between body composition parameters and treatment-related adverse events were evaluated using chi-square or Fisher’s exact tests, as appropriate. Because of the limited sample size and event count, multivariable logistic regression analyses for treatment-related adverse events were not performed in order to avoid model instability and overfitting.

A two-sided *p* value < 0.05 was considered statistically significant.

### 2.6. Ethical Considerations

The study was conducted in accordance with the Declaration of Helsinki and approved by the Research Ethics Committee for Medicines of Hospital Universitario de La Princesa (protocol code 4652). Written informed consent was obtained from all participants prior to enrolment in the study.

## 3. Results

### 3.1. Study Population and Baseline Characteristics

**Figure 1:**
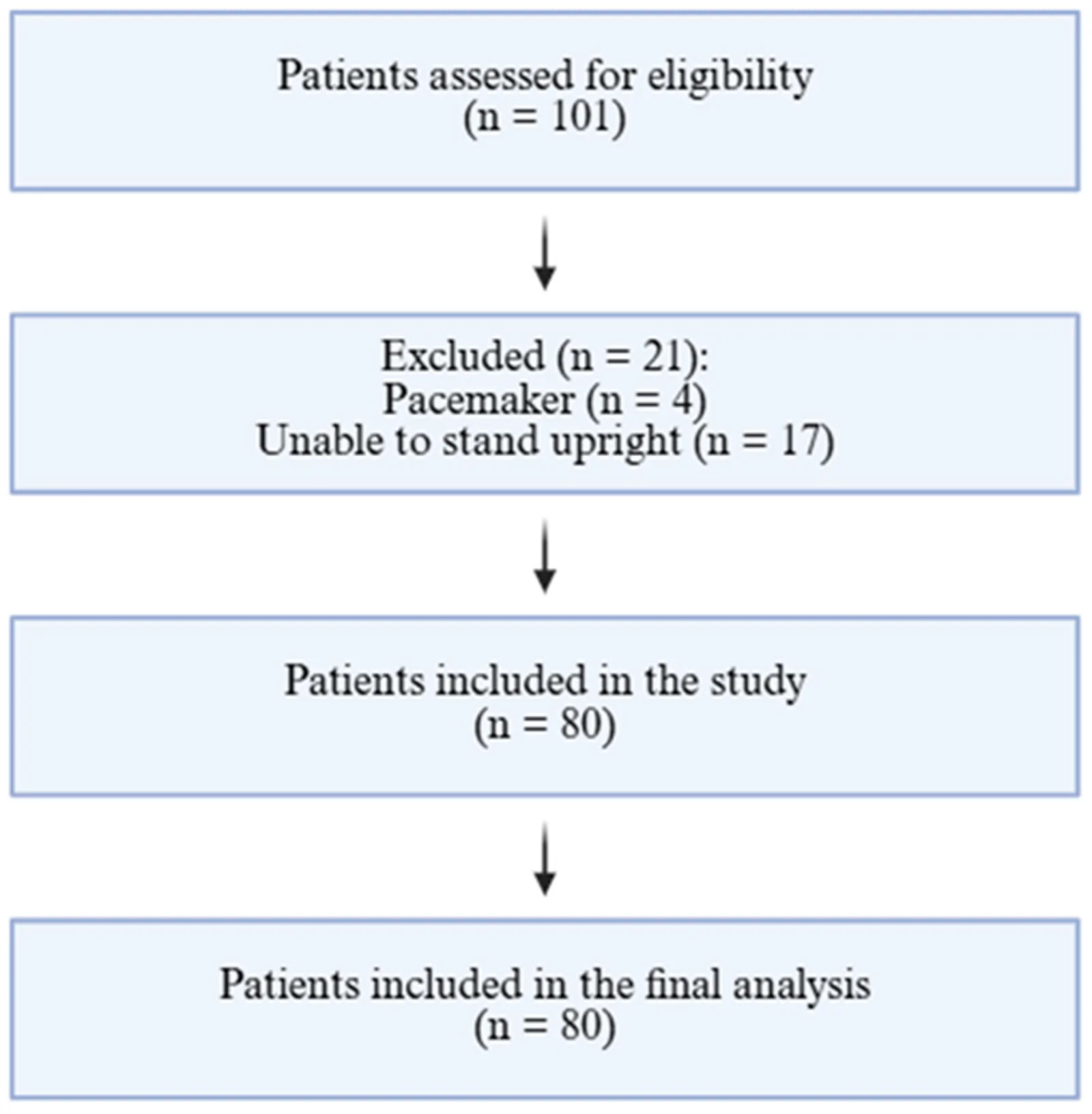
Flow diagram of the patient selection process and inclusion in the final analysis.

During the study period, 101 patients were assessed for eligibility (Figure 1). Of these, 21 were excluded: 4 because of pacemaker implantation and 17 because they were unable to maintain an upright standing position, precluding body composition assessment by BIA. Consequently, 80 patients met the inclusion criteria and were enrolled in the study. All enrolled patients completed the follow-up period and were included in the final analysis.

The cohort included a heterogeneous population of patients with different tumour types and immunotherapy regimens, reflecting routine clinical practice.

Baseline characteristics are summarised in Table 1. The median age at the initiation of treatment with PD-1/PD-L1 inhibitors was 67 years (range, 29–90 years), and 48 patients (60.0%) were male. Lung cancer was the most frequent tumour type (43/80, 53.75%), pembrolizumab was the most commonly administered PD-1/PD-L1 inhibitor (39/80, 48.75%), and 39 patients (48.75%) had stage IV disease at diagnosis. Detailed information on the immunotherapy regimen, treatment line, and monotherapy/combination status for each tumour type is provided in Supplementary Table S1.

### 3.2. Body Composition Characteristics

Baseline body composition characteristics are summarised in Table 2. Overall, the study population had a median BMI in the overweight range, with most patients presenting an elevated body fat percentage according to the predefined cut-off values.

### 3.3. Association Between BMI and Overall Survival

**Figure 2:**
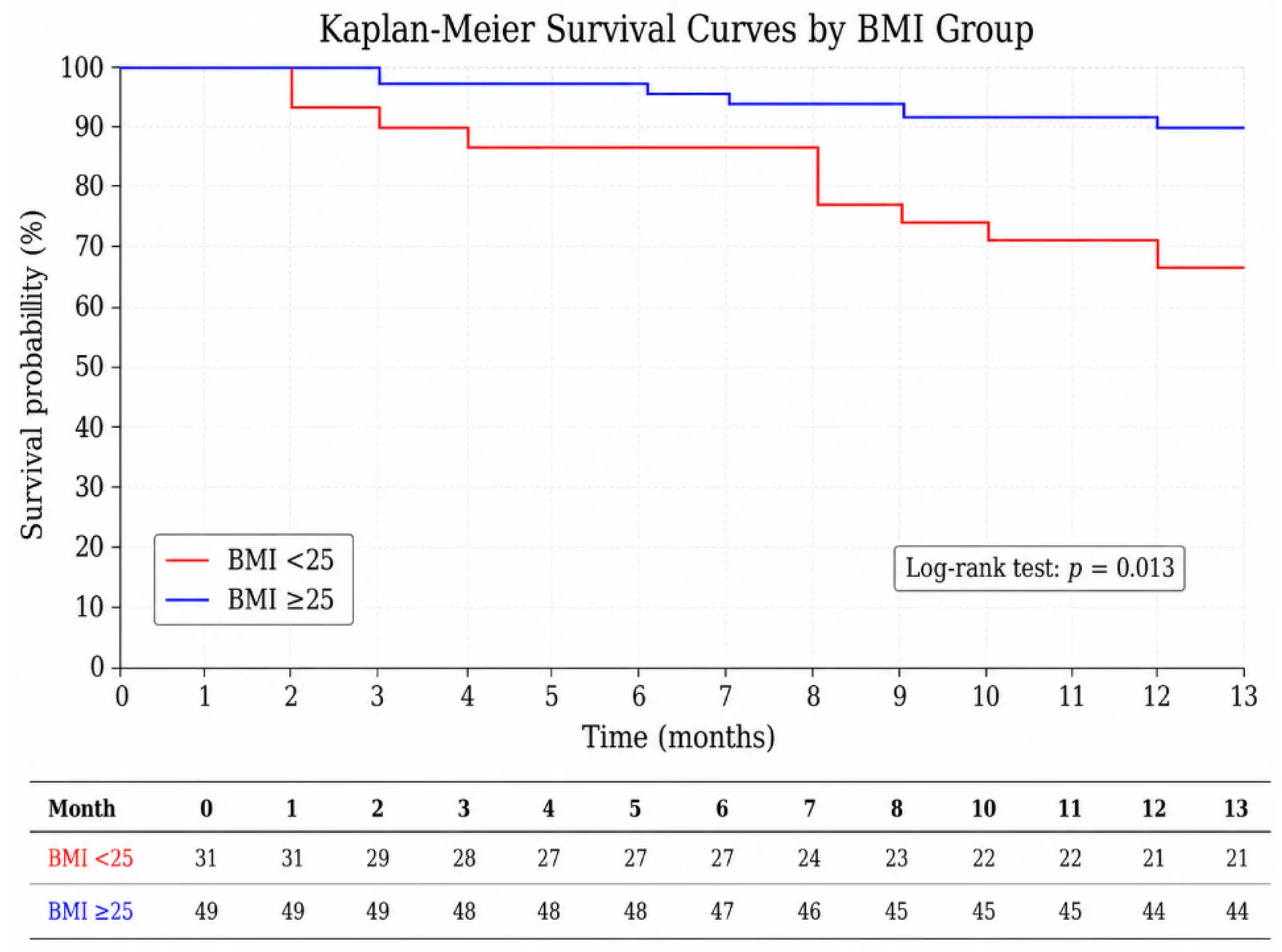
Kaplan–Meier overall survival curves according to BMI group. Group 0: BMI < 25 kg/m^2^. Group 1: BMI ≥ 25 kg/m^2^.

The association between BMI and overall survival was evaluated using Kaplan–Meier analysis and Cox proportional hazards regression. Patients with BMI ≥ 25 kg/m^2^ showed longer overall survival than those with BMI < 25 kg/m^2^ (Figure 2). In the multivariable Cox regression model adjusted for tumour stage and the presence of metastases, BMI ≥ 25 kg/m^2^ remained independently associated with a lower risk of death compared with BMI < 25 kg/m^2^ (HR 0.286, 95% CI 0.087–0.941; *p* = 0.039).

### 3.4. Association Between Body Fat Percentage and Overall Survival

**Figure 3:**
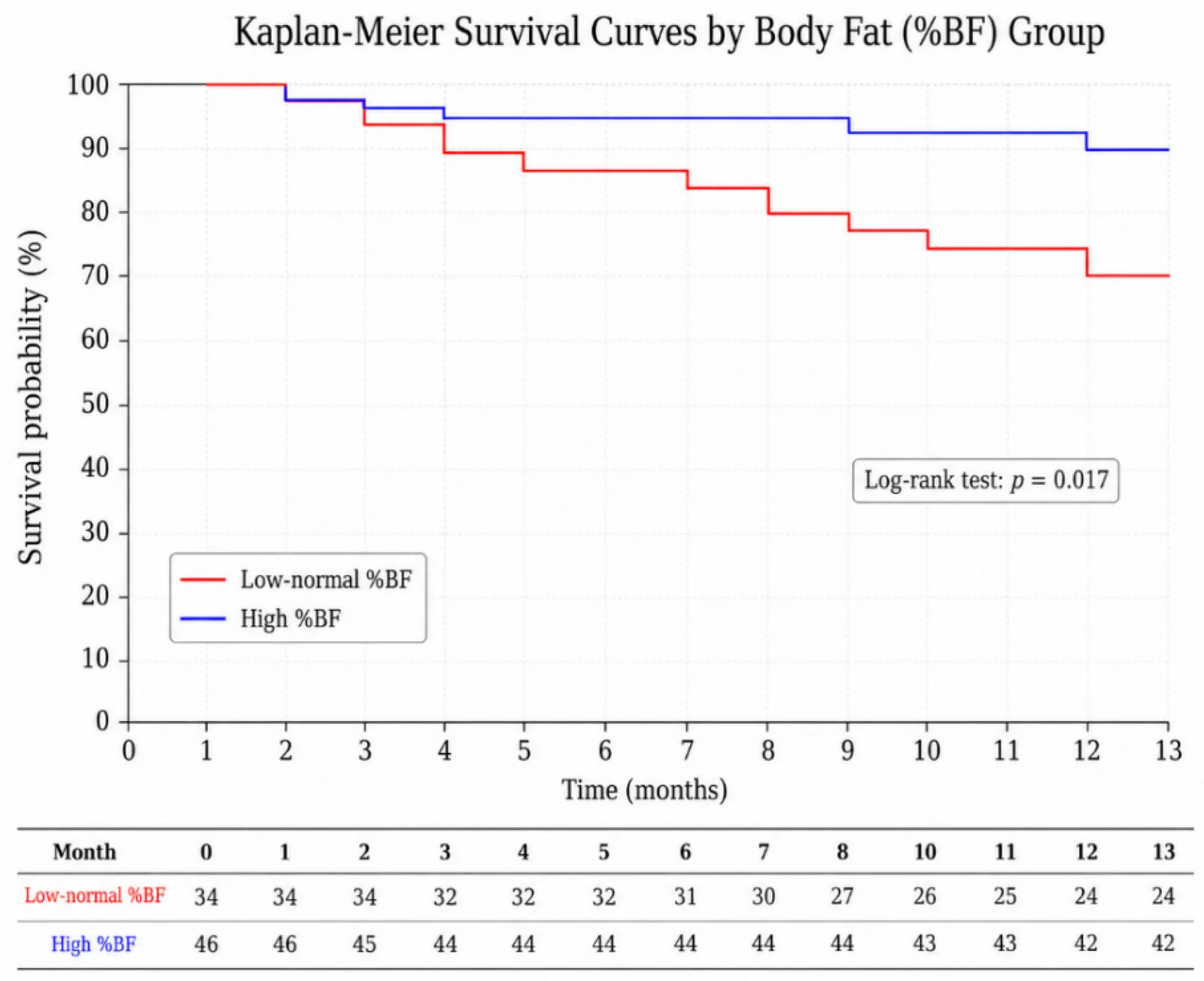
Kaplan–Meier curves for overall survival according to body fat percentage (%BF). Group 0: low/normal %BF. Group 1: high %BF.

The association between body fat percentage (%BF) and overall survival was evaluated using Kaplan–Meier analysis and Cox proportional hazards regression. Patients with elevated %BF showed longer overall survival than those with low or normal %BF (Figure 3). A trend toward an independent association between higher %BF and a lower risk of death was observed in the multivariable Cox regression model (HR 0.289, 95% CI 0.087–0.963; *p* = 0.036).

To explore whether body fat percentage (%BF) provided prognostic information beyond BMI, an additional multivariable Cox regression model including both variables simultaneously was performed. When BMI and %BF were entered into the same model, neither variable retained independent statistical significance. These findings suggest substantial overlap between both measures and indicate that they may represent related dimensions of adiposity rather than independent prognostic factors.

### 3.5. Association Between Body Composition and Progression-Free Survival

**Figure 4:**
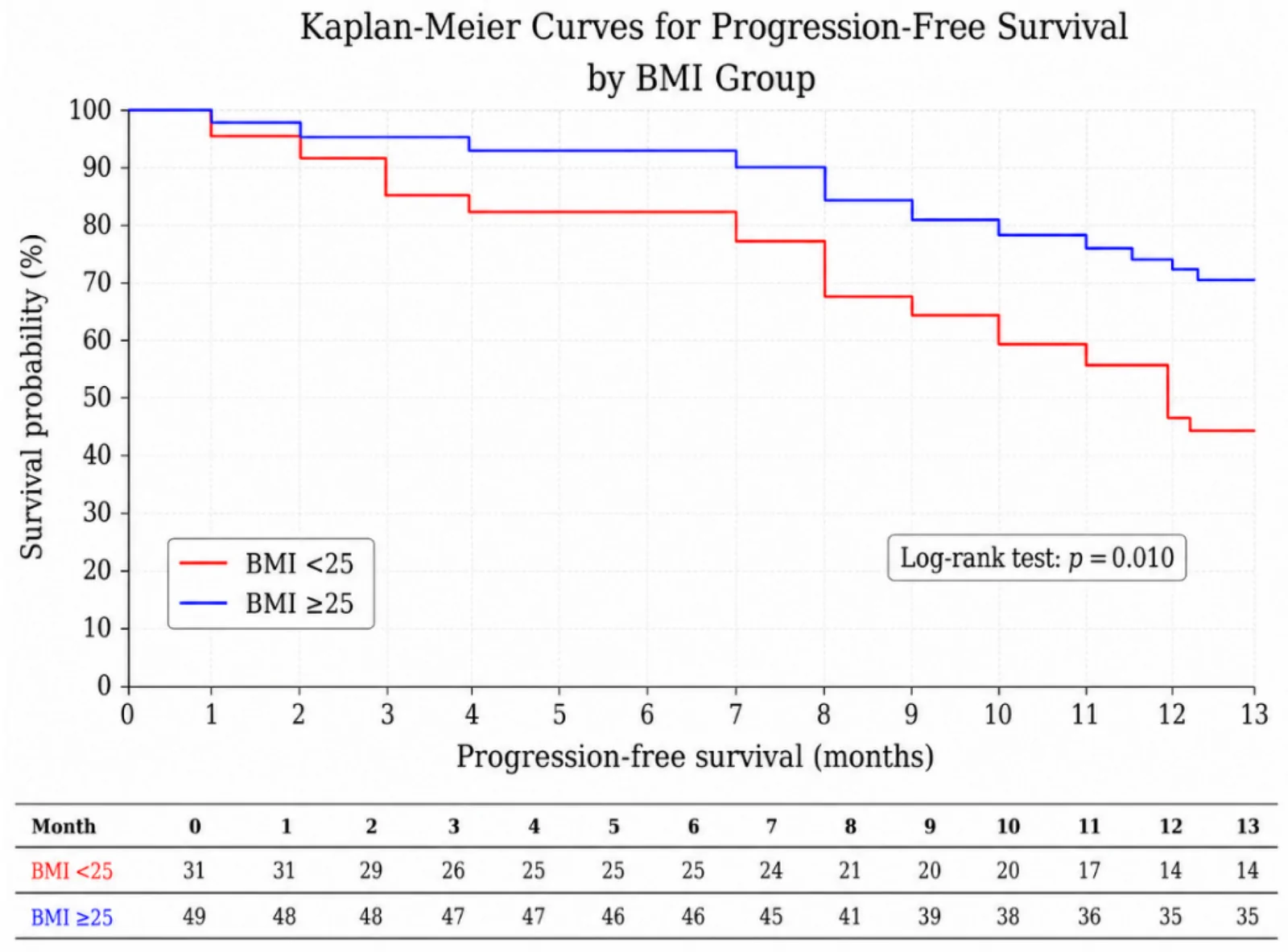
Kaplan–Meier progression-free survival curves according to BMI group. Group 0: BMI < 25 kg/m^2^. Group 1: BMI ≥ 25 kg/m^2^.

Progression-free survival was evaluated using the Kaplan–Meier method and the log-rank test. At 12 months, 31 progression events were recorded among the 80 patients (61.3% censored). Patients with BMI ≥ 25 kg/m^2^ had a longer mean PFS than those with BMI < 25 kg/m^2^ (37.3 vs. 25.9 months; log-rank *p* = 0.010), with an estimated 12-month PFS rate of 71.4% versus 45.2%, respectively (Figure 4).

**Figure 5:**
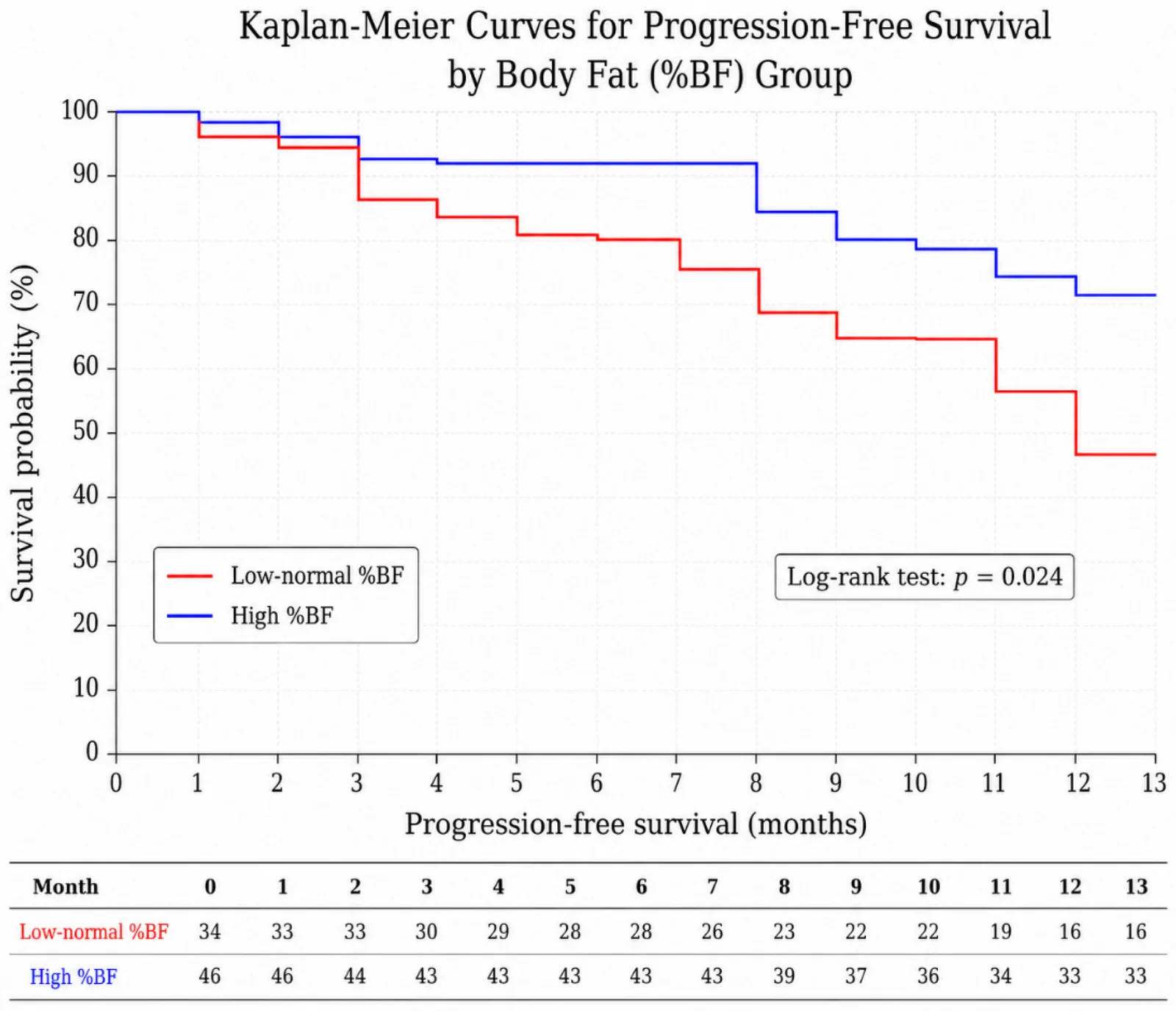
Kaplan–Meier progression-free survival curves according to body fat percentage (%BF). Group 0: low/normal %BF. Group 1: high %B.

Similar results were observed for %BF, with a longer mean PFS in patients with elevated %BF compared with those with low/normal %BF (37.4 vs. 26.8 months; log-rank *p* = 0.024) and a 12-month PFS rate of 71.7% versus 47.1%, respectively (Figure 5).

In a complementary exploratory analysis, patients who remained progression-free for at least 1 year showed a more adipose body composition profile than those who experienced disease progression within the first year (Table 3). Among patients with BMI ≥ 25 kg/m^2^, those who remained progression-free for at least 1 year had a higher median BMI than those who experienced progression within the first year (29.15 vs. 27.00 kg/m^2^). Similarly, among patients with elevated body fat percentage, those with prolonged PFS showed a higher median %BF than those with early progression (36.20% vs. 33.15%). These findings are consistent with the significant associations observed for BMI (*p* = 0.042) and %BF (*p* = 0.036). Body fat percentage also showed a positive correlation with 1-year progression-free survival (Pearson r = 0.39; Spearman ρ = 0.38). The remaining body composition parameters according to 1-year progression-free survival are presented in Table 3.

### 3.6. Combined Analysis of BMI and %BF

BMI and %BF were strongly correlated, both as continuous variables (Pearson r = 0.552; Spearman ρ = 0.550; both *p* < 0.001) and as their respective dichotomised categories (Pearson r = 0.614; Spearman ρ = 0.614; both *p* < 0.001), indicating substantial overlap between the two measures of adiposity.

When BMI category and %BF category were entered simultaneously into the same multivariable Cox regression model, adjusted for tumour stage and metastatic status, neither variable retained independent statistical significance for overall survival (BMI ≥ 25 kg/m^2^: HR 0.586, 95% CI 0.146–2.361, *p* = 0.453; elevated %BF: HR 0.352, 95% CI 0.079–1.566, *p* = 0.170) or for progression-free survival (BMI ≥ 25 kg/m^2^: HR 0.611, 95% CI 0.226–1.655, *p* = 0.333; elevated %BF: HR 0.699, 95% CI 0.255–1.913, *p* = 0.486). These findings suggest substantial overlap between both measures and indicate that they may represent related dimensions of adiposity rather than independent prognostic factors.

### 3.7. BMI and Adiposity Were Not Associated with the Occurrence of Immune-Related Adverse Events

Treatment-related adverse events were reviewed and reclassified according to their plausible immune-mediated origin. After this reclassification, 27 patients (33.8%) experienced at least one confirmed immune-related adverse event (irAE), accounting for 32 events in total (Table 4). The most frequent irAEs were pruritus (n = 9), nephrotoxicity (n = 8), arthritis (n = 4), immune-mediated diarrhoea (n = 3), hypothyroidism (n = 3), and hepatitis (n = 3), followed by encephalitis (n = 1) and neurotoxicity (n = 1). Of the 32 events, 21 (65.6%) were grade 1–2 and 11 (34.4%) were grade ≥3 according to CTCAE v5.0.

The association between body composition and the occurrence of confirmed irAEs was assessed using the chi-square test (Fisher’s exact test where expected cell counts were <5). No statistically significant association was observed between BMI category and irAE occurrence (χ^2^(1) = 1.43, *p* = 0.232; Fisher’s exact *p* = 0.332; Cramér’s V = 0.13) nor between %BF category and irAE occurrence (χ^2^(1) = 1.40, *p* = 0.237; Fisher’s exact *p* = 0.339; Cramér’s V = 0.13).

## 4. Discussion

In this study, we analysed whether body composition assessed by multifrequency bioelectrical impedance analysis (BIA) at treatment initiation was associated with survival outcomes and treatment-related toxicity in patients treated with PD-1/PD-L1 inhibitors.

The main findings suggest that a higher BMI was associated with prolonged one-year OS and PFS, while %BF showed a concordant survival pattern. In addition, patients with greater adiposity showed a more favourable 1-year progression-free survival profile. In contrast, neither BMI nor %BF was significantly associated with the occurrence of confirmed immune-related adverse events, suggesting that baseline body composition does not meaningfully predict irAE risk in this cohort.

These results are consistent with the so-called “obesity paradox”. Previous studies of patients with melanoma, lung cancer and renal cell carcinoma have reported longer OS and PFS for patients with a BMI ≥ 25 kg/m^2^ treated with PD-1/PD-L1 inhibitors than for underweight or normal-weight patients [16,17,18,19]. Our findings are consistent with these observations in a heterogeneous cohort of patients with solid tumours and extend previous evidence by incorporating BIA-derived body composition parameters.

Nevertheless, given the exploratory nature of this pilot study, these results should be interpreted as hypothesis-generating rather than definitive.

A critical consideration in interpreting our data is the possibility of reverse causality. A lower BMI or %BF at baseline might not only represent a lack of “protective” adiposity but could instead be a clinical manifestation of cancer cachexia, a more aggressive disease biology, or a poorer functional status. Patients with lower weight often present with higher systemic inflammation and depleted nutritional reserves, which are established negative prognostic factors in oncology. Therefore, the association observed between higher adiposity and improved outcomes should not be interpreted as evidence of a direct beneficial effect of obesity itself. The biological and clinical interpretation of the obesity paradox remains complex and is likely influenced by multiple host-, tumour-, and treatment-related factors [16,17].

The observed association may be explained by several mechanisms. Adipose tissue acts as an endocrine and immunologically active organ that secretes adipokines and proinflammatory cytokines such as leptin, IL-6 and TNF-α, which are capable of modulating T-cell activation and proliferation [18,19,20]. Studies using preclinical models have linked obesity to a state of chronic low-grade inflammation and increased PD-1 expression in T lymphocytes, which could increase sensitivity to immune checkpoint inhibitors [21]. Consequently, excess adiposity may promote a tumour microenvironment that is more susceptible to the therapeutic effects of PD-1/PD-L1 inhibitors, potentially amplifying their antitumour activity.

Among the mechanisms involved, hyperleptinaemia, a characteristic of obesity, deserves particular attention, as it exerts significant immunomodulatory effects by influencing the activation, differentiation and function of T lymphocytes. Emerging evidence suggests that leptin may modulate PD-1 expression and signalling, forming a leptin–PD-1 axis that could partially explain the improved responses observed in patients with greater adiposity. However, these mechanisms remain under investigation, and the relationships among obesity, inflammation and the immune response remain complex [22,23,24].

A relevant contribution of this study is the incorporation of BIA-derived body composition variables into the analysis. Unlike most previous studies, which have relied exclusively on BMI—an indirect measure which does not distinguish between fat mass and lean mass—BIA provides a more detailed morphologic analysis of nutritional status [13,25]. In our cohort, %BF, classified according to the Gallagher criteria, showed a survival pattern that was concordant with BMI, particularly for PFS, suggesting that adiposity, rather than total body weight alone, may play a modulatory role in the response to immunotherapy [14,26]. However, when BMI and %BF were entered simultaneously into the same multivariable Cox model, neither retained independent statistical significance. This finding suggests substantial overlap between these measures and indicates that they may reflect the same underlying biological construct of adiposity rather than independent prognostic factors. Therefore, the present study does not support the conclusion that %BF provides prognostic information beyond that obtained from BMI alone.

Unlike the initial exploratory analysis based on overall treatment-related adverse events, the reclassified analysis restricted to confirmed immune-related adverse events did not show a significant association between BMI, %BF and irAE occurrence in our cohort. The absence of association with confirmed irAEs should be interpreted cautiously given the limited number of events and the relatively small number of grade ≥3 toxicities. These findings differ from some previous studies reporting a higher frequency of immune-related adverse events in patients with obesity receiving immune checkpoint inhibitors [27,28,29,30,31].

Furthermore, the descriptive nature of the toxicity analysis and the small number of events do not allow conclusions regarding specific toxicity patterns according to BMI or body fat categories. Therefore, these toxicity findings should be considered exploratory and interpreted with caution rather than as evidence of a causal relationship between adiposity and treatment-related toxicity. Further prospective studies are needed to clarify the relationship between body composition, treatment-related adverse events, and clinical outcomes in patients receiving PD-1/PD-L1 inhibitors.

Our study has several limitations. First, the relatively small sample size, the limited number of survival and progression events, and the single-centre design limit the generalisability of the findings. These factors limited the complexity of multivariable modelling, increased the risk of model overfitting, and precluded more robust time-to-event analyses for progression-free survival. Therefore, the observed associations should be interpreted cautiously and considered hypothesis-generating until confirmed in larger prospective cohorts with a higher number of outcome events.

Second, the tumour and therapeutic heterogeneity of the cohort may introduce uncontrolled confounding factors; however, it also enhances the applicability of the findings across a broad range of tumour types. Although lung cancer represented more than half of the study population, the limited sample size precluded adequately powered tumour-specific subgroup analyses, including analyses restricted to lung cancer. In addition, important prognostic variables such as PD-L1 expression, ECOG performance status, treatment line, disease burden, concurrent systemic therapies, and previous treatments were not systematically available for all patients and therefore could not be incorporated into the multivariable analyses. Consequently, residual confounding cannot be excluded and may have influenced both survival outcomes and body composition measures.

Third, the toxicity analysis was exploratory in nature. Not all treatment-related adverse events could be unequivocally classified as immune-mediated, and the limited number of grade ≥3 events prevented robust analyses according to toxicity severity.

Finally, although bioelectrical impedance analysis is an accessible and non-invasive tool, its accuracy may be influenced by hydration status and other uncontrolled variables. Moreover, body composition was assessed only at baseline, and longitudinal changes could not be evaluated. In addition, the observational design limits causal inference, and residual confounding and reverse causality cannot be excluded.

Despite these limitations, this study provides additional real-world evidence in an area where prospective data remain limited, particularly in the Spanish population setting, and supports the hypothesis that adiposity-related host factors may be associated with both the efficacy and safety of immunotherapy. However, the observational nature of the study precludes causal inference, and the findings should be considered exploratory and hypothesis-generating. Therefore, our findings support further investigation of body composition assessment as a potential prognostic tool in patients receiving PD-1/PD-L1 inhibitors. Future studies should focus on larger tumour-specific prospective cohorts, incorporate longitudinal body composition assessments, and account for established prognostic biomarkers and clinical variables, including PD-L1 expression and performance status, to determine whether body composition provides prognostic information beyond conventional predictors of immunotherapy outcomes.

## 5. Conclusions

In this prospective exploratory study, higher BMI and elevated body fat percentage (%BF) at treatment initiation were independently associated with more favourable one-year overall survival, and both were also associated with improved progression-free survival in patients with solid tumours receiving PD-1/PD-L1 inhibitors. When BMI and %BF were analysed together in the same multivariable Cox model, neither retained independent statistical significance, indicating that they capture overlapping rather than independent prognostic information and likely reflect a shared underlying construct of adiposity. In contrast, neither BMI nor %BF was associated with the occurrence of confirmed immune-related adverse events, suggesting that baseline body composition does not meaningfully predict treatment-related toxicity risk in this cohort.

Given the observational design, limited sample size, low number of outcome events, and clinical heterogeneity of the cohort, these findings should be considered exploratory and hypothesis-generating rather than definitive, and the present study does not demonstrate a causal effect of adiposity on the efficacy or safety of PD-1/PD-L1 inhibitors.

Further prospective studies incorporating larger tumour-specific cohorts, longitudinal body composition assessment, and established clinical and molecular prognostic factors are required to determine whether body composition provides prognostic information beyond conventional predictors of immunotherapy outcomes. Future research should also clarify whether bioelectrical impedance analysis-derived parameters provide additional prognostic value beyond BMI and whether body composition assessment may have a role in patient stratification during immunotherapy.

## Figures and Tables

**Table 1:** Baseline demographic and oncological characteristics.

**Demographic characteristics**	
Characteristic	Overall cohort (n = 80)
Age, median (IQR), years	67 (29–90)
Male sex, n (%)	48 (60.0)
Female sex, n (%)	32 (40.0)
**Oncological characteristics**	
Tumour type	n (%)
Lung	43 (53.75)
Urothelial	13 (16.25)
Liver	6 (7.50)
Breast	4 (5.00)
Head and neck	4 (5.00)
Gastric	4 (5.00)
Melanoma	3 (3.75)
Colon	2 (2.50)
Thymus	1 (1.25)
**Treatment**	
Immunotherapy received	n (%)
Pembrolizumab	39 (48.75)
Atezolizumab	18 (22.50)
Nivolumab	12 (15.00)
Durvalumab	11 (13.75)
**Tumour stage at diagnosis**	
Stage type	n (%)
Stage I	4 (5.00)
Stage II	7 (8.75)
Stage III	26 (32.50)
Stage IV	39 (48.75)

**Table 2:** Baseline Body Composition Characteristics.

BODY COMPOSITION				
Variable	Category	n	Me	IQR
Skeletal muscle mass (kg)	Total	80	28.40	11.90
	Low-normal	65	25.80	10.70
	High	15	36.70	6.85
Body fat percentage (%)	Total	80	31.20	18.72
	Low-normal	34	21.50	10.43
	High	46	35.70	8.50
Bone mass (kg)	Total	80	3.04	0.98
	Low-normal	43	2.91	0.86
	High	37	3.08	1.05
Extracellular water (L)	Total	80	14.70	5.53
	Low-normal	51	12.65	4.10
	High	29	17.50	2.50
Intracellular water (L)	Total	80	23.30	9.22
	Low-normal	65	21.30	8.20
	High	15	29.70	5.30
BMI (kg/m^2^)	Total	80	26.05	6.62
	Underweight	3	17.40	1.95
	Normal weight	28	22.50	1.90
	Overweight	30	26.80	1.93
	Obesity	19	32.10	5.75
BMI (kg/m^2^)	<25	31	22.40	2.75
	≥25	49	28.50	4.70

Me: Median; IQR: Interquartile Range.

**Table 3:** Body composition characteristics and adiposity-related measures according to 1-year progression-free survival.

Variable	Category	PFS < 1 Year (n = 59) Median (IQR)	PFS ≥ 1 Year (n = 21) Median (IQR)	Total (n = 80) Median (IQR)	* *p*-Value
Skeletal muscle mass (kg)	Low-normal	28.50 (10.37)	25.20 (10.20)	25.80 (10.70)	
	High	40.50 (7.10)	35.30 (4.57)	36.70 (6.85)	0.523
	Total	30.50 (15.00)	27.40 (11.25)	28.40 (12.10)	
Body fat percentage (%)	Low-normal	20.30 (7.65)	23.60 (10.90)	21.50 (10.43)	
	High	33.15 (13.53)	36.20 (7.97)	35.70 (8.50)	0.036
	Total	24.50 (13.40)	32.20 (12.80)	31.20 (13.72)	
Bone mass (kg)	Low-normal	2.69 (0.83)	2.57 (0.62)	2.57 (0.63)	
	High	3.89 (0.47)	3.40 (0.88)	3.56 (1.00)	0.332
	Total	3.24 (1.29)	3.01 (0.93)	3.04 (0.98)	
Extracellular water (L)	Low-normal	14.70 (5.35)	12.55 (3.65)	12.60 (5.10)	
	High	21.25 (3.12)	16.80 (3.65)	17.50 (2.50)	0.599
	Total	16.50 (7.50)	14.50 (5.25)	14.70 (5.53)	
Intracellular water (L)	Low-normal	23.40 (7.95)	20.90 (7.90)	21.30 (8.20)	
	High	32.60 (5.50)	28.55 (3.57)	29.70 (5.30)	0.579
	Total	24.90 (11.50)	22.60 (8.55)	23.30 (8.32)	
BMI (kg/m^2^)	<25 kg/m^2^	21.90 (3.43)	22.50 (1.70)	22.40 (2.75)	
	≥25 kg/m^2^	27.00 (7.15)	29.15 (4.05)	28.50 (4.70)	0.042
	Total	25.00 (5.00)	26.40 (6.80)	26.05 (6.62)	

* *p*-values refer to comparisons between the predefined adiposity categories (BMI < 25 vs. ≥25 kg/m^2^ and low/normal vs. high body fat percentage groups) according to 1-year progression-free survival status.

**Table 4:** Frequency and distribution of confirmed immune-related adverse events (irAEs).

Adverse Event	n (%) ^1^	Grade 1–2	Grade ≥ 3
Nephrotoxicity	8 (10.0)	1	7
Immune-mediated diarrhoea	3 (3.8)	2	1
Hypothyroidism	3 (3.8)	3	0
Arthritis	4 (5.0)	4	0
Pruritus	9 (11.3)	9	0
Encephalitis	1 (1.3)	0	1
Hepatitis	3 (3.8)	2	1
Neurotoxicity	1 (1.3)	0	1
Total irAEs	32	21 (65.6) ^2^	11 (34.4) ^2^
Patients with ≥1 irAE	27 (33.8)	—	—

^1^ Percentage calculated over the total cohort (N = 80). ^2^ Percentage calculated over the total number of confirmed irAE events (n = 32). Severity graded according to CTCAE v5.0.

## Data Availability

The data presented in this study are available from the corresponding author upon reasonable request. The data are not publicly available due to ethical and privacy restrictions, as they contain information that could compromise the confidentiality of study participants.

## Supplementary Materials

The following supporting information can be downloaded at: https://www.mdpi.com/article/10.3390/jpm16090457/s1, Table S1. Distribution of immunotherapy drug, treatment line, and monotherapy/combination status by tumour type (N = 80).

## Abbreviations

The following abbreviations are used in this manuscript:

%BF	Body Fat Percentage
BIA	Bioelectrical Impedance Analysis
BMI	Body Mass Index
CI	Confidence Interval
ICIs	Immune Checkpoint Inhibitors
IQR	Interquartile Range
CTCAE	Common Terminology Criteria for Adverse Events
HR	Hazard Ratio
Me	Median
OS	Overall Survival
PD-1	Programmed Cell Death Protein-1
PD-L1	Programmed Death-Ligand 1
PFS	Progression-Free Survival
SD	Standard Deviation
