# Supplementary material for: Body Mass Index and Body Fat Percentage Are Associated with Clinical Outcomes in Patients Receiving PD-1/PD-L1 Inhibitors: A Prospective Exploratory Study
Source: J Pers Med. 2026 Aug 30;16(9):457. doi: 10.3390/jpm16090457 (PMC13608239; doi:10.3390/jpm16090457)
Supplement: Supplementary file 1 [file jpm-16-00457-s001.zip › jpm-4484840-supplementary.pdf]

Table S1. Distribution of immunotherapy drug, treatment line, and monotherapy/combination status by tumour type (N = 80).

Tumour Type	n	Drug	Line	Monotherapy/Combination
**Lung**	4	Atezolizumab	First	Combination
	4	Atezolizumab	Second	Monotherapy
	1	Atezolizumab	Second	Combination
	1	Durvalumab	First	Monotherapy
	10	Durvalumab	Second	Monotherapy
	1	Nivolumab	Second	Combination
	10	Pembrolizumab	First	Monotherapy
	10	Pembrolizumab	First	Combination
	1	Pembrolizumab	Second	Monotherapy
	1	Pembrolizumab	Second	Combination
**Urothelial**	1	Atezolizumab	First	Monotherapy
	1	Atezolizumab	Second	Monotherapy
	2	Nivolumab	First	Monotherapy
	1	Nivolumab	First	Combination
	2	Nivolumab	Second	Monotherapy
	3	Pembrolizumab	First	Monotherapy
	2	Pembrolizumab	First	Combination
	1	Pembrolizumab	Second	Monotherapy
**Liver**	6	Atezolizumab	First	Combination
**Breast**	1	Atezolizumab	First	Combination
	2	Pembrolizumab	First	Combination
	1	Pembrolizumab	Second	Combination
**Head and neck**	3	Nivolumab	Second	Monotherapy
	1	Pembrolizumab	First	Combination
**Gastric**	1	Nivolumab	Second	Monotherapy
	1	Nivolumab	Second	Combination
	2	Pembrolizumab	Second	Combination
**Melanoma**	1	Nivolumab	First	Monotherapy
	2	Pembrolizumab	First	Monotherapy
**Colon**	1	Pembrolizumab	Second	Monotherapy

Tumour Type	n	Drug	Line	Monotherapy/Combination
	1	Pembrolizumab	Second	Combination
**Thymus**	1	Pembrolizumab	Second	Combination

Note: totals by drug across the cohort were Atezolizumab, n = 18; Nivolumab, n = 12; Durvalumab, n = 11; Pembrolizumab, n = 39, consistent with Table 1.
